# Reliable measurements of brain atrophy in individual patients with multiple sclerosis

**DOI:** 10.1002/brb3.518

**Published:** 2016-07-19

**Authors:** Dirk Smeets, Annemie Ribbens, Diana M. Sima, Melissa Cambron, Dana Horakova, Saurabh Jain, Anke Maertens, Eline Van Vlierberghe, Vasilis Terzopoulos, Anne‐Marie Van Binst, Manuela Vaneckova, Jan Krasensky, Tomas Uher, Zdenek Seidl, Jacques De Keyser, Guy Nagels, Johan De Mey, Eva Havrdova, Wim Van Hecke

**Affiliations:** ^1^ R&DicometrixLeuvenBelgium; ^2^ BioImaging LabUniversiteit AntwerpenAntwerpBelgium; ^3^ Department of NeurologyCenter for NeurosciencesUniversitair Ziekenhuis Brussel, Vrije Universiteit Brussel (VUB)BrusselBelgium; ^4^ Department of Neurology and Center of Clinical Neuroscience Charles University in PragueFirst Faculty of Medicine and General University HospitalPragueCzech Republic; ^5^ Department of Radiology 1st Faculty of Medicine and General University HospitalCharles UniversityPragueCzech Republic; ^6^ National Multiple Sclerosis CentrumMelsbroekBelgium

**Keywords:** brain atrophy, magnetic resonance images, MSmetrix, multiple sclerosis

## Abstract

**Introduction:**

As neurodegeneration is recognized as a major contributor to disability in multiple sclerosis (MS), brain atrophy quantification could have a high added value in clinical practice to assess treatment efficacy and disease progression, provided that it has a sufficiently low measurement error to draw meaningful conclusions for an individual patient.

**Method:**

In this paper, we present an automated longitudinal method based on Jacobian integration for measuring whole‐brain and gray matter atrophy based on anatomical magnetic resonance images (MRI), named MS**metrix**. MS**metrix** is specifically designed to measure atrophy in patients with MS, by including iterative lesion segmentation and lesion filling based on FLAIR and T1‐weighted MRI scans.

**Results:**

MS **metrix** is compared with SIENA with respect to test–retest error and consistency, resulting in an average test–retest error on an MS data set of 0.13% (MS **metrix**) and 0.17% (SIENA) and a consistency error of 0.07% (MS **metrix**) and 0.05% (SIENA). On a healthy subject data set including physiological variability the test–retest is 0.19% (MS **metrix**) and 0.31% (SIENA).

**Conclusion:**

Therefore, we can conclude that MS**metrix** could be of added value in clinical practice for the follow‐up of treatment and disease progression in MS patients.

## 1 Introduction

Classically, multiple sclerosis (MS) has been regarded as an autoimmune disease of the white matter (WM) in the central nervous system leading to severe disability over the course of several decades. The pathological hallmark of MS is the presence of focal areas of demyelination of the brain and spinal cord WM. Over the years, substantial evidence emerged that gray matter (GM) is also heavily affected and that neurodegenerative phenomena such as neuronal/axonal damage and whole‐brain and GM atrophy play an important role in MS (Friese, Schattling, & Fugger, 2014; Trapp & Nave, 2008). Although neurodegeneration has been considered as a secondary phenomenon to inflammation and demyelination, studies have demonstrated that neurodegeneration develops along with inflammation and demyelination (Dutta & Trapp, 2007; Silber & Sharief, 1999). In addition, it has been shown that the neurodegenerative component of MS is responsible for the irreversible disability and is prognostic for short‐ and long‐term disability and cognitive decline (Bjartmar, Kidd, Mörk, Rudick, & Trapp, 2000).

In recent years, magnetic resonance imaging (MRI) brain scans have been increasingly used to measure brain atrophy in MS patients. Typically, a volumetric T1‐weighted MRI data set is used to calculate the whole‐brain, WM, GM, or cerebrospinal fluid (CSF) volume or atrophy. It has been demonstrated that brain atrophy, as measured on MRI scans, is already present in patients with a clinically isolated syndrome suggestive of MS and in patients with early (<5 years from diagnosis) definite MS (Chard et al., 2002; Chard & Miller, 2009; Henry et al., 2008; Raz et al., 2010). In addition, it has been shown that brain atrophy occurs in all types of MS, relapsing‐remitting MS (RRMS), primary and secondary progressive MS (PPMS and SPMS) (De Stefano et al., 2010; Tedeschi et al., 2005). In contrast to WM atrophy, which is relatively constant across all disease stages, GM atrophy has been correlated with disease progression, physical disability, and cognitive impairment (Amato et al., 2004; Chard et al., 2004; Sanfilipo, Benedict, Sharma, Weinstock‐Guttman, & Bakshi, 2005; Sanfilipo, Benedict, Weinstock‐Guttman, & Bakshi, 2006; De Stefano et al., 2003).

As neurodegeneration is recognized as an important aspect of MS and a major contributor to disability in MS, brain atrophy has been increasingly used in clinical trials as an outcome measure to assess treatment efficacy and disease progression. Studies with conventional therapies such as interferon‐β and glatiramer acetate have shown only limited effects on brain atrophy (Filippi et al., 2004; Leary & Thompson, 2003; Molyneux et al., 2000). However, the FREEDOMS study in patients with RRMS demonstrated that fingolimod significantly reduced brain atrophy over 2 years, compared with placebo (Radue et al., 2012; Silber et al., 2014). In addition, it was reported in the TRANSFORMS study that MS patients on fingolimod had a lower brain atrophy rate compared to patients using intramuscular interferon‐β‐1a (Cohen et al., 2013). Decreases in brain atrophy in RRMS patients have also been reported with laquinimod treatment (Comi et al., 2012). Brown and Coles (2013) noted decreased brain atrophy in patients treated with alemtuzumab compared to interferon‐β for RRMS, while Portaccio et al. (2013) had similar findings of lower brain atrophy in patients treated with natalizumab compared to interferon‐β.

An additional advantage of measuring brain atrophy in clinical trials is that 10 times less subjects need to be included, when the trial is powered on an outcome of a 50% reduction in MRI lesions and atrophy, compared to using disability endpoints (Polman et al., 2006; Sormani et al., 2001; Sormani, Arnold, & De Stefano, 2014; De Stefano et al., 2010).

Despite the importance of neurodegeneration in MS patients and the known positive effect of some drugs on slowing down this neurodegeneration, brain atrophy is currently not used in clinical practice to assess individual MS patients. As MS is a heterogeneous disease with a relatively unpredictable course, there is a clear need for objective measures that can be evaluated for individual patients and used for follow‐up and treatment decisions. Several measures for disability are used, such as the Expanded Disability Status Score and the MS Functional Composite scale (Cutter et al., 1999; Kurtzke, 1983). However, it is known that they can be unpredictable within the same patient, being characterized by phases with predominant occurrence of relapses versus progression. Although neurodegeneration is only one of many aspects of the disease, a reliable measure of brain atrophy on individual MS patients will help in making informed decisions and moving into a more personalized and evidence‐based medicine in MS.

Techniques to measure brain volume or brain volume loss can be subdivided into two main categories, that is, cross‐sectional and longitudinal methods (Giorgio, Battaglini, Smith, & De Stefano, 2008; De Stefano et al., 2014). Cross‐sectional methods use a single MRI scan to segment specific tissues or structures. As a result, the volume of these tissue types and/or structures is calculated. Well‐known and validated examples of these are BPF (Rudick, Fisher, Lee, Simon, & Jacobs, 1999), SIENAX (Smith et al., 2002, 2004), and Freesurfer (Fischl et al., 2002). In contrast to cross‐sectional approaches, longitudinal methods take into account two MRI scans of the same subject from different time points to calculate brain volume changes or atrophy. Longitudinal methods typically try to match the two MRI scans using warping techniques and directly extract small changes in brain volume from this process (e.g., Boyes et al., 2006; Freeborough & Fox, 1997; Smith, De Stefano, Jenkinson, & Matthews, 2001). A longitudinal method that is frequently used in clinical trials (see, e.g., Comi et al., 2013) is SIENA (Smith et al., 2001), while a longitudinal processing pipeline that can take more than two time points into account is included in FreeSurfer (Reuter, Schmansky, Rosas, & Fischl, 2012).

When applying brain atrophy measures for individual MS patients, the measurement error of the method and thus the reliability becomes of paramount importance. It is indeed known that the average atrophy rate in MS patients is approximately 0.5%–1.3% per year, compared with 0.1%–0.4% per year in healthy individuals (Barkhof, Calabresi, Miller, & Reingold, 2009; Fotenos, Mintun, Snyder, Morris, & Buckner, 2008; Simon, 2006). The measurement error of the brain atrophy measure therefore needs to be very low, in order to draw meaningful conclusions in individual patients. This includes a robustness of the method toward daily physiological processes that might affect brain volume.

In this paper, an automated longitudinal registration‐based method is proposed to measure whole‐brain and GM atrophy by performing Jacobian integration within the segmentations, with the Jacobian extracted from the registration. The longitudinal method is initialized by a cross‐sectional method providing lesion filled images as well as the segmentations. In order to assess the method's future applicability for atrophy quantification of individual MS patients, the paper is focused on the reliability of the method. The reliability of the method will be evaluated in terms of measurement error, robustness toward physiological processes and consistency.

## 2 Materials and Methods

### 2.1 MRI acquisition

#### 2.1.1 Data set 1

Data set 1 was acquired from 10 MS patients who participated in a study at the University Hospital Brussels, Belgium. The study was approved by the local ethics committee and all patients signed informed consent forms. MR imaging was performed for each patient twice on 3T whole body scanners from three different manufacturers (GE Medical Systems Discovery MR750w; SIEMENS Skyra; Philips Medical Systems Achieva). Subjects were repositioned on the scanner console between the two scans, and therefore all scans can be treated as individual measurements. A total of 27 test–retest scan pairs (9 Philips, 10 Siemens, 8 GE) was obtained as scan sessions were sometimes missed by patients.

The MRI protocol consists each time of a 3D T1‐weighted image and a 3D FLAIR sequence. The GE scanner protocol contained, among others, two 3D sequences: a fat‐saturated 3D FLAIR (TR 9,500 ms, TE 135.78 ms, TI 2,428.0 ms, 240 × 240 mm^2^ field of view (FOV), 232 sagittal slices, 0.4688 × 0.4688 × 0.7 mm^3^ voxel resolution) and a 3D T1‐weighted FSPGR sequence (TR 7.32 ms, TE 3.144 ms, FA 12°, 220 × 220 mm^2^ FOV, 328 sagittal slices, 0.4297 × 0.4297 × 0.5 mm^3^ voxel resolution). The SIEMENS scanner protocol contained, among others, two 3D sequences: a fat‐saturated 3D FLAIR (TR 5,000 ms, TE 387.0 ms, TI 1,800.0 ms, 230 × 230 mm^2^ FOV, 192 sagittal slices, 0.4492 × 0.4492 × 0.9 mm^3^ voxel resolution) and a 3D T1‐weighted MPRAGE sequence (TR 2,300 ms, TE 2.29 ms, FA 8°, 240 × 240 mm^2^ FOV, 176 sagittal slices, 0.9375 × 0.9375 × 0.94 mm^3^ voxel resolution). The PHILIPS scanner protocol contained, among others, two 3D sequences: a fat‐saturated 3D FLAIR (TR 4,800 ms, 240 × 240 mm^2^ FOV, 321 sagittal slices, 1.0416 × 1.0416 × 0.56 mm^3^ voxel resolution) and a 3D T1‐weighted FSPGR sequence (TR 4.936 ms, FA 8°, 230 × 230 mm^2^ FOV, 310 sagittal slices, 0.5324 × 0.5324 × 0.5 mm^3^ voxel resolution). The resolution of T1‐weighted and FLAIR images from all the scanners is high and therefore, due to very high computational memory requirement, all T1‐weighted images were downsampled to (1.0 × 1.0 × 1.0 mm^3^) resolution. The FLAIR image was not downsampled at this point because it is rigidly registered to T1‐weighted image in the initial stage of the method and thus will have the (downsampled) T1‐weighted image resolution.

#### 2.1.2 Data set 2

Data set 2 is the publicly available data set described in Maclaren, Han, Vos, Fischbein, and Bammer (2014). They acquired the data set with the approval of the Stanford University Institutional Review Board and all subjects gave their written informed consent.

A total of 120 T1‐weighted images were acquired from three healthy subjects (40 scans/subject). Each subject was scanned two times on 20 different days within a 31‐day period. Subjects were repositioned on the scanner console between the two scans in each session, so that all scans were treated as separate measurements (with a resulting break of ~5 min between scans). All images are acquired on the GE MR750 3T scanner using the ADNI‐recommended T1‐weighted imaging protocol for this system (accelerated sagittal 3D IR‐SPGR, standard 8‐channel phased array head coil, TR 7.3 ms, TE 3 ms, TI 400 ms, FA 11°, 256 × 256 matrix slice, 270 mm FOV, 1.2 mm slice thickness, acquisition time: 5 min 37 s).

#### 2.1.3 Data set 3

Data set 3 was available from the department of radiology of the General University Hospital in Prague and consists of brain MR images obtained from 20 subjects. All patients signed informed consent forms.

Each subject was scanned on a 1.5T scanner (Gyroscan, Philips Medical Systems, Best, the Netherlands) at months 0, 6, 12, and 24 using each time the same imaging protocol. Axial brain images were acquired using fast fluid‐attenuated inversion recovery (FLAIR; TR 11,000 ms, TE 140 ms, TI 2,600 ms, FA 90°, 56 × 181 mm^2^ FOV, 1 × 1 × 1 mm^3^ voxel resolution) and T1‐weighted three‐dimensional fast field echo images (TR 25 ms, TE 5 ms, FA 30°, 256 × 256 mm^2^ FOV, 1 × 1 × 1 mm^3^ voxel resolution).

### 2.2 MRI analysis

#### 2.2.1 MSmetrix

**Figure 1:**
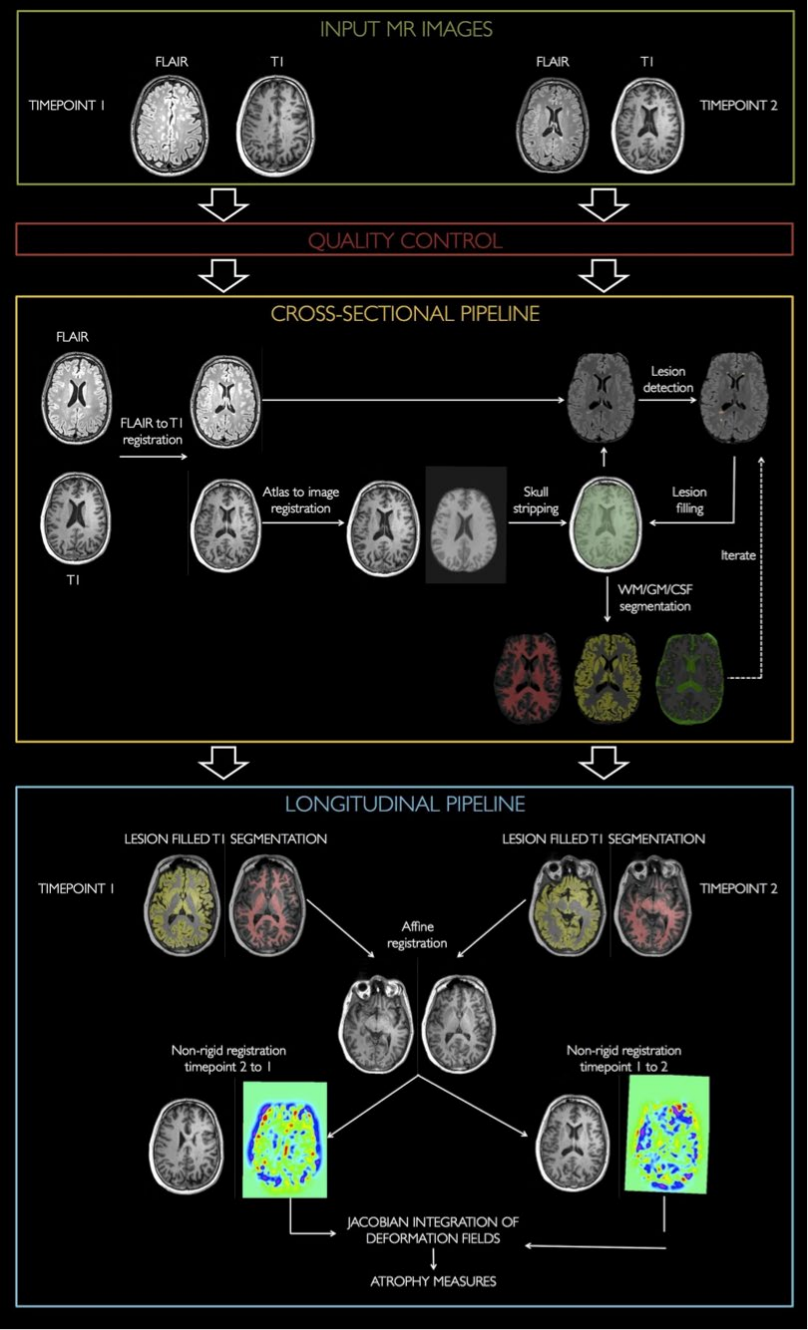
Schematic overview of MS **metrix**‐long. MS **metrix**‐long starts with a quality control of the images. Subsequently, MS **metrix**‐cross is performed for each time point. The results from the cross‐sectional step are used to initialize the longitudinal pipeline. In the longitudinal step, the brain atrophy is calculated based on a Jacobian integration in both directions

The proposed method, for which an overview is shown in Fig. 1, starts with a 3D FLAIR and 3D T1‐weighted MR image for each of the time points. Each time point is processed independently by a cross‐sectional pipeline (MS**metrix** ‐cross) that computes in a fully automated way segmentations of WM, GM, and CSF and produces T1‐weighted images that are bias corrected, lesion filled, and skull stripped. Subsequently, the longitudinal pipeline is executed.

##### **MS**metrix‐cross

MS**metrix**‐cross (Jain et al., 2015) is a cross‐sectional pipeline and hence handles the images of each time point separately. This automated pipeline is initialized by some preprocessing steps before entering the main loop of the algorithm. These preprocessing steps include: (1) matching the FLAIR image with the T1‐weighted image, (2) skull stripping by transferring a brain mask from the MNI atlas toward the T1‐weighted image, (3) warping probabilistic anatomical priors for GM, WM, and CSF toward the T1‐weighted image space. All preprocessing steps require an affine registration (Ourselin, Roche, Subsol, Pennec, & Ayache, 2001), and in step (2) and (3), a nonrigid registration is performed as well (Modat et al., 2014).

The main loop of the algorithm iterates over (1) the segmentation of the T1‐weighted image into GM, WM, and CSF, including bias correction, (2) outlier detection on the FLAIR images, (3) extraction of the lesions from the outliers, and (4) filling of the lesions in the T1‐weighted image (Jain et al., 2015).

##### **MS**metrix‐long

The longitudinal method starts from a T1‐weighted and a FLAIR image at two time points and consists of four fully automated steps.

First, the cross‐sectional pipeline (MS**metrix**‐cross) is executed, generating lesion filled and bias corrected T1‐weighted images for each time point (Jain et al., 2015), and their segmentation into WM, GM, and CSF.

In the second step, the T1‐weighted images of the two time points are affinely registered, both from the first to the second as from the second to the first time point (i.e., two registrations in both directions are executed). The affine registration consists of (1) a rigid registration based on the whole image, (2) an affine registration based on the skull to compensate for small scaling differences due to distortions, and (3) a rigid registration on the whole brain in order to correct for small translation and rotation errors in the skull based co‐registration. The rigid and/or affine registration is based on a block matching approach (Ourselin et al., 2001). To improve the robustness of each co‐registration, a symmetric approach (Modat et al., 2014) is used, which imposes, in theory, that the transformations are diffeomorphic. For the skull image, required in step (2), the region around the brain is used. This region is computed by binary dilating the brain mask, available from the cross‐sectional pipeline, with a kernel of 20 mm and subsequently subtracting the brain mask.

In the third step, the affinely registered and bias‐corrected T1‐weighted images are nonrigidly registered. This registration is again performed in both directions, respectively, from the first to the second and from the second to the first time point. The nonrigid registration (Modat et al., 2014) uses normalized mutual information (NMI) as similarity and B‐splines to restrict the deformation, with a final grid spacing of two voxels. The registration algorithm makes use of a multiresolution approach with two levels (grid space 4 and 2 mm, respectively) to allow sufficiently large deformation in order to capture the expected maximal atrophy between the time points (±5%). Furthermore, an additional penalty term is added for the Jacobian determinant of the deformation field, which slightly penalizes large local variations in the deformation field. In this way, we ensure plausible deformation fields. Thus, the objective function is based on the NMI similarity measure and a log of the Jacobian determinant penalty term with weight = 0.95 for the former and 0.05 for the latter term.

In the fourth and final step, the percentage volume change between the scans is calculated based on a Jacobian integration of the segmentations. The Jacobian determinant represents local shrinkage (for values < 1) or expansion (for values > 1) and can be calculated from the deformation field describing the nonrigid registration. Here, the Jacobian determinant is calculated for each nonrigid registration (i.e., from time point 1 to time point 2 and vice versa) and subsequently integrated over the segmentations (summation in this area) of the corresponding time point. This results in volume estimations of the tissue classes of the other time point, after a correction for the voxel size.

The percentage change of GM and parenchymal (GM + WM) volume can now be calculated in both directions based on the original volume of time point 1 (resp. time point 2) and the volume of time point 2 (resp. time point 1) obtained from the Jacobian integration time point 2). Finally, the percentage change in both directions is averaged to obtain the final percentage change of GM parenchymal volume.

#### 2.2.2 SIENA(X)

SIENA and SIENAX are part of the FMRIB Software Library (http://www.fmrib.ox.ac.uk/fsl), and refer to the longitudinal brain atrophy and cross‐sectional brain volume measurement, respectively (Smith et al., 2001, 2002, 2004; Zhang, Brady, & Smith, 2001). The input images for both SIENAX and SIENA are T1‐weighted MRI data sets.

##### SIENAX

The pipeline is initialized by applying the Brain Extraction Tool (BET; Smith, 2002), which creates a brain mask in three steps: (1) global intensity thresholding to roughly select brain from non‐brain region; (2) tessellated spherical mask creation, positioned at the approximate center of gravity of the brain; (3) iterative refinement toward the brain's edge, using smoothness criteria and a local intensity threshold. Subsequently, voxels within the obtained brain mask are classified in several classes, depending on the image intensities. As a result, CSF, WM, GM, and background are segmented, and the cross‐sectional volumes can be obtained, referred to as SIENAX (Zhang et al., 2001).

##### SIENA

SIENA is initialized by applying the BET generating a brain mask for both images (Smith, 2002). Subsequently, an explicit skull image is extracted from the images (Jenkinson, Bannister, Brady, & Smith, 2002; Jenkinson & Smith, 2001). The images are then warped into an intermediate space where the skull image is used to guide the scaling. The time point 1 image in the intermediate space is segmented (analogously to SIENAX) and the edge between the brain parenchyma (WM + GM) and CSF is determined. Subsequently, the brain parenchyma/CSF edge displacement between the two time points is estimated by aligning the peaks of the spatial derivatives of the intensity profiles of both images. Finally, the mean edge displacement is converted into a global estimate of percentage brain volume change (PBVC) between the two time points.

In this study, SIENA(X) was run with no manual correction and with the default parameters, except for the ‘‐B’' option for the brain extraction tool BET, which was modified from its default parameter *f* = 0.5 to the value *f* = 0.1, as found optimal in Popescu et al. (2012).

#### 2.2.3 Validation

MS**metrix**‐cross/long and SIENA(X) are evaluated in terms of reliability of the atrophy measurements for individual patients. To this end, a validation is performed in terms of the measurement error, the robustness toward physiological changes and the consistency of the method. Per experiment, significant difference between MS**metrix**‐cross/long and SIENA(X) will be evaluated using the parametric paired *t*‐test as well as the nonparametric Wilcoxon signed‐rank test at significance level 0.01.

First, we quantify the measurement error based on test–retest images from MS patients (data set 1). The test–retest images are acquired on the same scanner and the same day. The measurement error for atrophy is computed as the estimated percentage volume change of the parenchymal volume and of GM on these pairs of images, which is expected to be zero.

Subsequently, the robustness of the method is tested in terms of physiological changes (data set 2). MR images acquired at two successive time points from the same healthy subject are considered as test and retest scans, regardless of the exact time interval (0–3 days). Hence, this evaluation captures the measurement error as well as robustness to daily changes due to physiological processes.

Finally, the consistency of the atrophy measurements over time is evaluated (data set 3). Atrophy measurements are performed for MR images from MS patients acquired with time gap of at least 6 months. For any three consecutive time points, *T*1*, T*2*, T*3, with 6 months gap, the consistency index (CI) is computed as the absolute difference in PBVC between *T*1 and *T*3 on one hand and the sum of the PBVC between *T*1 and *T*2 and between *T*2 and *T*3 on the other hand*,* that is, $\text{CI}=|{\text{PBVC}}_{\left(T1-T3\right)}-\left({\text{PBVC}}_{\left(T1-T2\right)}+{\text{PBVC}}_{\left(T2-T3\right)}\right)|$, This is motivated by linearly approximating the direct atrophy measurement from *T*1 to *T*3 with the cumulative atrophy measurement from *T*1 to *T*3 via *T*2.

## 3 Results

### 3.1 Test–retest measurement error

After visual quality control of the test–retest images (data set 1), four images were exhibiting artifacts, thus four image pairs are removed from the data set, resulting in, respectively, 23 image pairs (7 Philips, 8 Siemens, and 8 GE).

**Figure 2:**
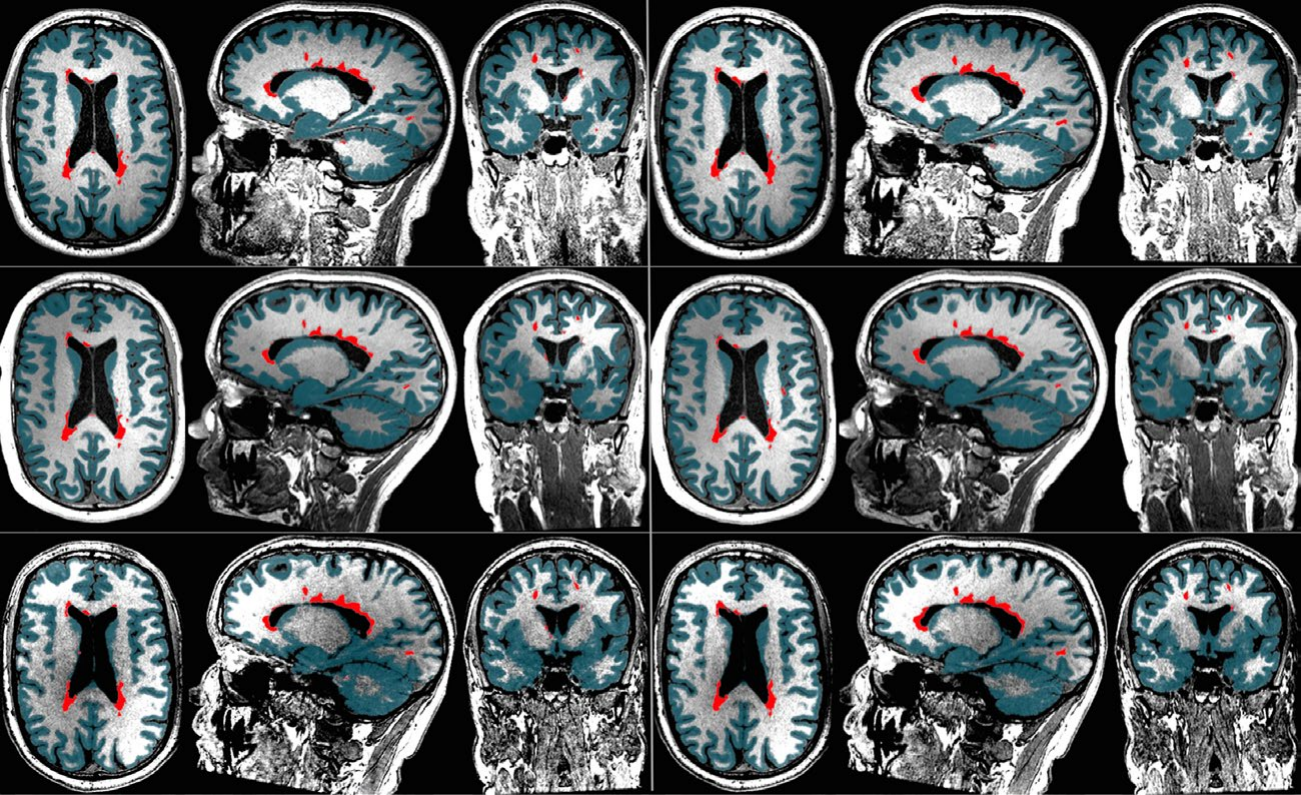
Illustration of MS **metrix**‐cross on six repeated scans of the same MS patient (data set 1). Each row shows test and retest segmentations for Philips (top), Siemens (middle) and GE (bottom). Lesions are marked with red and GM segmentation with teal

**Figure 3:**
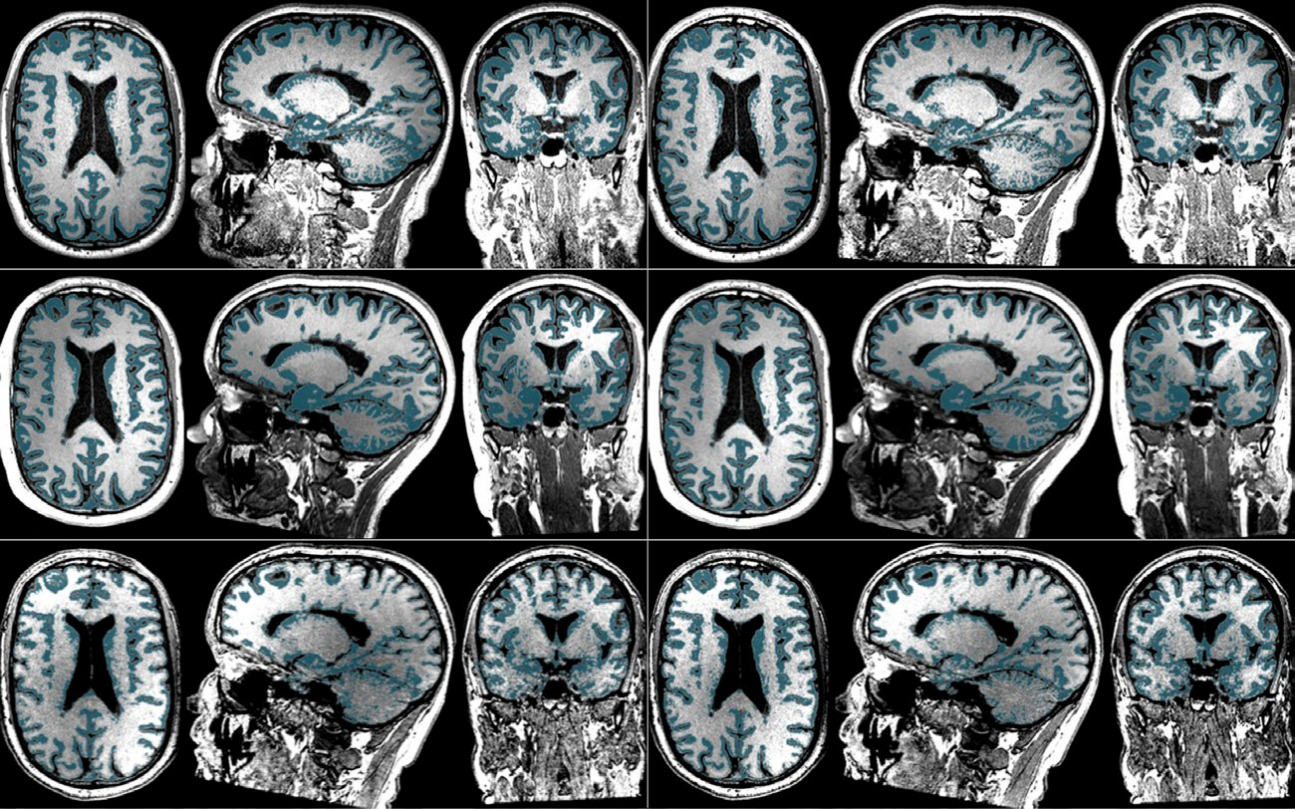
Illustration of SIENAX on six repeated scans of the same MS patient (data set 1). Each row shows test and retest segmentations for Philips (top), Siemens (middle), and GE (bottom). GM segmentation is marked with teal

Figures 2 and 3 show an illustration of the test–retest segmentations on six repeated scans of the same MS patient obtained with MS**metrix**‐cross and SIENAX, respectively. These segmentations also form the base of the longitudinal methods. MS**metrix** consistently finds the same WM lesions in all scans. It can be observed that the lesions are typically labeled as GM by SIENAX, since it does not include lesion segmentation.

**Figure 4:**
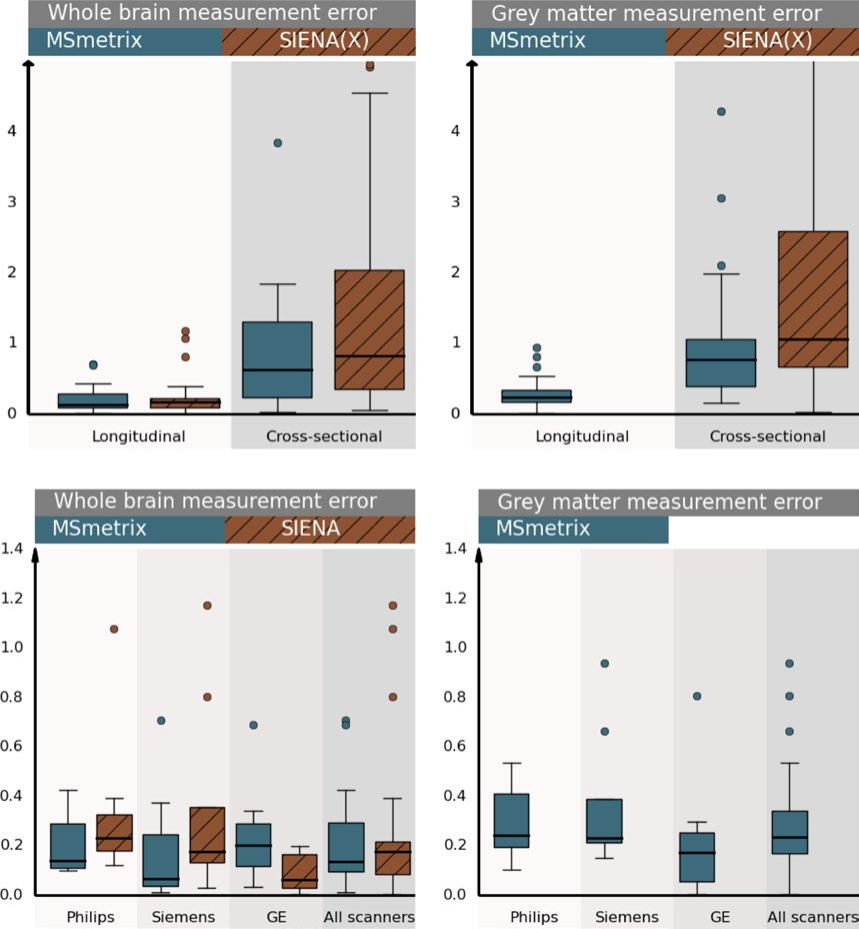
First row: Comparison of the measurement error of the longitudinal and cross‐sectional methods MS **metrix** and SIENA(X) on test–retest scans from all MS patients in data set 1. Boxplots show absolute values of the whole brain (left) and gray matter (right) percentual volume change, computed either by the longitudinal approaches or based on two cross‐sectional measurements on the test–retest scans. Second row: Per‐scanner comparison of the measurement error of the longitudinal methods MS **metrix** and SIENA on test–retest scans from all MS patients in data set 1. Boxplots show absolute values of the whole brain (left) and gray matter (right) percentual volume change

In Fig. 4 the measurement error of the cross‐sectional methods is compared with those of the longitudinal methods on MS patients (data set 1), indicating the necessity of longitudinal measurements for reliable atrophy quantifications. Moreover, MS**metrix** shows measurement errors comparable to or lower than SIENA(X). The test–retest percentage whole‐brain volume changes computed by MS**metrix**‐long differ in absolute value from the expected 0% by 0.13% (median over all scan pairs, on all three scanners; first and third quartiles: 0.09–0.29%, maximum value: 0.7%), while those of SIENA differ from 0 in absolute value by 0.17% (first and third quartiles: 0.08–0.22%, maximum value: 1.2%). However, the difference between MS**metrix**‐long and SIENA is not significant (*p* = .54 for the paired *t*‐test and *p* = .60 for the Wilcoxon signed‐rank test). For the cross‐sectional methods, the median percentage whole‐brain volume change error is 0.62% (first and third quartiles: 0.23–1.3%, maximum value: 3.8%) for MS**metrix**‐cross and 0.82% (first and third quartiles: 0.34–2.04%, maximum value: 6.8%) for SIENAX. Also, the cross‐sectional methods MS**metrix**‐cross and SIENAX are not significantly different (*p* = .10 for the paired *t*‐test and *p* = .16 for the Wilcoxon signed‐rank). However, MS**metrix**‐long is significantly different from the cross‐sectional methods, that is MS**metrix**‐cross and SIENAX (*p* < .01 for the paired *t*‐test and for the Wilcoxon signed‐rank test).

The measurement error for GM atrophy is also quantified, except for SIENA, as GM atrophy is not provided by SIENA. MS**metrix**‐long differs from the expected 0% absolute percentage GM volume change with 0.23% (first and third quartiles: 0.17–0.34%, maximum value: 0.9%), MS**metrix**‐cross with 0.77% (first and third quartiles: 0.39–1.06%, maximum value: 4.3%), while SIENAX with 1.06% (first and third quartiles: 0.67–2.59%, maximum value: 11.1%). Significance testing showed that MS**metrix**‐long is significantly different from MS**metrix**‐cross and from SIENAX (*p* < .01 for both the paired *t*‐test and the Wilcoxon signed‐rank test). The measurement error of GM atrophy for MS**metrix**‐cross is significantly smaller than SIENAX at significance level 0.05, but not at significance level 0.01 (*p* = .03 for the paired *t*‐test and *p* = .02 for the Wilcoxon signed‐rank test).

For completeness, the measurement errors for whole‐brain and GM atrophy are also shown per scanner for the longitudinal methods (Fig. 4). No significant differences were observed between MS**metrix**‐long and SIENA for each of the scanners (paired *t*‐test, Wilcoxon signed‐rank test, significance level 0.01).

### 3.2 Robustness towards physiological processes

**Figure 5:**
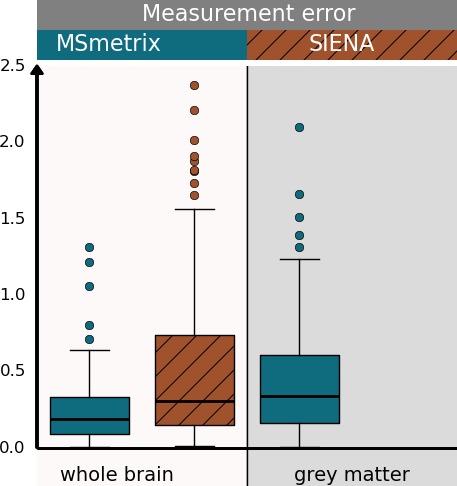
Boxplots of absolute percentual volume change (whole brain and gray matter) on successive scans from healthy subjects (time interval < 3 days) (data set 2)

Figure 5 summarizes the absolute percentage volume changes on pairs of successive scans of healthy subjects (data set 2). All scans are acquired within the same month and successive scans have a maximum time interval of 3 days. The percentage whole‐brain volume changes differ in median absolute value from the expected 0% by 0.19% for MS**metrix**‐long (first and third quartiles: 0.09–0.33%, maximum value: 1.3%), while SIENA has a median absolute deviation from 0 of 0.31% (first and third quartiles: 0.15–0.74%, maximum value: 2.4%). For GM, MS**metrix**‐long has a median absolute deviation from 0 of 0.34% (first and third quartiles: 0.16–0.61%, maximum value: 2.1%). Both the parametric paired *t*‐test and the nonparametric Wilcoxon signed‐rank test show significant differences in absolute percentage whole‐brain volume changes between MS**metrix** and SIENA (*p* < .01).

### 3.3 Longitudinal consistency

**Figure 6:**
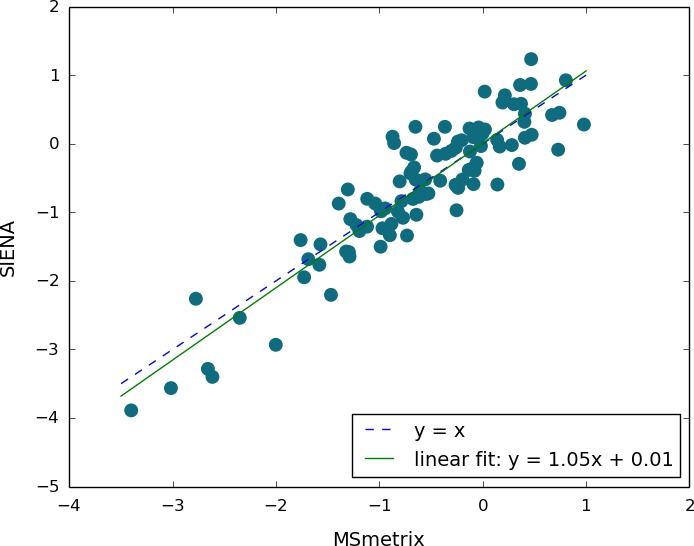
Comparison of whole‐brain percentual volume change obtained by MS **metrix**‐long and SIENA in 20 MS patients, five time points each

On a longitudinal data set of patients with MS (data set 3), the correlation between whole‐brain atrophy measurements obtained with MS**metrix**‐long and SIENA is relatively high, with a Pearson correlation coefficient equal to 0.91 and an intraclass correlation coefficient of 0.90. Figure 6 presents the scatter plot of the percentage whole‐brain volume changes of MS**metrix**‐long with respect to SIENA's for 6‐months, 1‐year, and 2‐year atrophy for all 20 patients.

The CI for 6‐month intervals compared to the 1‐year interval of the whole‐brain percentage volume change had a median absolute value of 0.07% for MS**metrix**‐long (first and third quartiles: 0.04–0.11%, maximum value: 0.15%) and 0.05% for SIENA (first and third quartiles: 0.02–0.08%, maximum value: 0.17%). No significant difference was observed for the CI between MS**metrix**‐long and SIENA (*p* = .42 for the paired *t*‐test and *p* = .35 for the Wilcoxon signed‐rank test). For GM, the CI was 0.13% for MS**metrix**‐long (first and third quartiles: 0.09–0.23%, maximum value: 0.39%).

## 4 Discussion

In this manuscript, an automated longitudinal Jacobian integration‐based method for measuring whole‐brain and GM atrophy is introduced. In order to assess the use of this method in clinical practice on MRI data sets of individual MS patients, the reliability of the method is evaluated in terms of the method's measurement error, of its robustness toward physiological processes, and of its longitudinal consistency. Results were compared to SIENA, a well‐validated method that is commonly used for measuring brain atrophy in clinical studies and trials. Note that only whole‐brain atrophy results are compared with SIENA, as the software does not provide GM atrophy measurements.

The MS**metrix** software pipeline is specifically designed to measure atrophy in patients with MS, by including iterative lesion segmentation and lesion filling based on FLAIR and T1‐weighted MRI scans. In this context, it is known that applying brain volume measures without performing lesion filling can introduce errors between 0.3% and 2.5%, depending on the lesion size and lesion intensity (Battaglini, Jenkinson, & De Stefano, 2012; Chard, Jackson, Miller, & Wheeler‐Kingshott, 2010; Popescu et al., 2014).

When brain atrophy measures are introduced in clinical practice for individual MS patients, interpretation of these results should be done with caution. In this context, it is indeed known that there are many confounding factors that can affect the measurement of brain atrophy and therefore the interpretation of the results (Bermel & Bakshi, 2006; Simon, 2006; Zivadinov & Minagar, 2009). For example, it is known that brain volume changes are not only caused by neuronal or axonal loss but that also demyelination and inflammation can play a role (Giorgio et al., 2008). In addition, brain volume loss as measured using MRI is affected by the use of steroids or some disease modifying therapies. It has indeed been demonstrated that their anti‐inflammatory properties decrease the brain volume in the first 6 months to 1 year of treatment, typically referred to as pseudoatrophy (Zivadinov et al., 2008). In this context, it was suggested that the measurement of GM volume loss is less susceptible to this pseudoatrophy compared to whole‐brain or WM volume changes (Nakamura, Fox, & Fisher, 2011; Tiberio et al., 2005).

Since the difference between brain atrophy in MS patients (0.5%–1.3% yearly atrophy) and healthy subjects (0.1%–0.4% yearly atrophy) is small, and clinicians would like to assess if an individual patient is stable on brain atrophy or not, a small measurement error is of paramount importance to draw meaningful conclusions in clinical practice (Barkhof et al., 2009; Fotenos et al., 2008; Simon, 2006).

This paper focuses on the reliability of the methods on MRI data sets from MS patients acquired using a “clinical” MRI protocol. In order to introduce brain atrophy measures in clinical practice, acceptable measurement and reproducibility errors are required on MRI scans that can be obtained in a clinical setting with a limited acquisition time.

In contrast to most other studies that have investigated such errors of brain atrophy measures, for this paper, repeat scans were acquired on patients with MS instead of on healthy subjects (Cover et al., 2011; Maclaren et al., 2014; Nakamura et al., 2014; Smith et al., 2001). As a result, the errors presented in this paper can be seen as representative for a clinical setting for patients with MS.

MS**metrix**‐long results on data set 1 demonstrated a small measurement error across the three 3T scanners, with a median value of 0.13% over all scanners. These errors are within the tolerance level that might be attributed to normal variations in healthy controls, but are lower than the expected atrophy levels in pathology. Although the results of SIENA were not significantly different based on a parametric and nonparametric statistical test (*p* > .05), a larger median value (0.17%) over all scanners was observed for SIENA. This error can change when using different parameter settings of SIENA, for example, for this data set, the median absolute error was double if we kept the default parameter values. We tried to use optimal settings, as were described in the literature (Popescu et al., 2012).

Note that is has been demonstrated in the past that using cross‐sectional methods to measure atrophy results in much higher errors compared to longitudinal approaches (Durand‐Dubief et al., 2012; Nakamura et al., 2014; Smith et al., 2002). Our results confirm these findings. The measurement errors for the cross‐sectional methods were significantly higher than those of the longitudinal methods (*p* < .01 for both parametric and nonparametric tests).

The test–retest error for whole‐brain and GM atrophy computed by MS**metrix**‐cross was lower than for SIENAX. These relatively lower measurement error for MS**metrix**‐cross compared to SIENAX might contribute to the lower values of MS**metrix**‐long compared to SIENA, as they are used as input data. The reported values for SIENA and SIENAX are similar to that observed by Smith et al. (2001, 2002), even though scans in their study were obtained from healthy subjects.

In addition to the measurement error, robustness toward daily physiological processes is evaluated using data set 2, where MS**metrix**‐long still results in a small overall error for whole‐brain atrophy, while SIENA shows a significant larger error compared to MS**metrix**‐long. A median absolute value of 0.19% was observed for MS**metrix**‐long and of 0.31% for SIENA for whole‐brain atrophy. For GM, the median absolute value is 0.23% for MS**metrix**‐long. This indicates that MS**metrix**‐long is more robust toward daily physiological effects than SIENA.

Finally, the consistency of the methods is assessed using data set 3. No significant differences were observed between MS**metrix**‐long and SIENA in terms of the CI for 6‐month intervals compared to the 1‐year interval of the whole‐brain atrophy.

It is important to notice that in addition to small errors, including measurement errors, robustness toward daily physiological processes and consistency, the brain atrophy software should still be sensitive enough to detect small changes. This can be evaluated on longitudinal MRI data of MS patients. Although there is no ground truth available of the exact changes in brain atrophy that should be detected, our results suggest that MS**metrix** has a high correlation with SIENA, which has already been used as surrogate outcome measure in several MS clinical trials. Also, the sensitivity of MS**metrix**‐long has been demonstrated in other domains, that is, the detection of dehydration effects and separating healthy controls from Alzheimer patients (Ribbens et al., 2015). In view of the measurement errors reported in this paper, one should consider as potentially pathological change any whole‐brain atrophy levels exceeding, for example, 0.7%–1% per year.

In this work, we have also shown that GM atrophy can be measured automatically alongside whole‐brain atrophy using MS**metrix**‐long. Although the absolute measurement errors are higher than those for whole brain, the approach still has potential to detect GM atrophy reliably if this is abnormally large and should prompt immediate therapy re‐evaluation (e.g., >1.5% per year).

Note that the results on GM atrophy measurement were not directly compared against another longitudinal technique in this paper. The widely used SIENA does not return GM atrophy. Other approaches for longitudinal atrophy computations are, like SIENA, not specifically designed for MS, and thus lesion filling is often recommended as a preprocessing step before applying these methods. For instance, the longitudinal brain segmentation pipeline (Reuter et al., 2012) included in FreeSurfer offers the possibility to compute volume changes of brain substructures, after transforming multiple time point images to a common subject‐specific template space. In MS patients, a disadvantage might be that large deviations between the individual time points (e.g., large atrophy or the presence of large new lesions) might have an unpredictable effect on the template creation or might bias the results of individual time points toward the common template.

We could conclude that due to the low measurement error, MS**metrix**‐long could be of added value to the clinical practice for the follow‐up of treatment and disease progression in MS patients.

## Funding Information

Seventh Framework Programme (Grant/Award Number: ‘FP7‐COOPERATION‐2013‐602150’, ‘FP7‐PEOPLE‐2013‐IAPP‐612360’, ‘FP7‐PEOPLE‐2012‐ITN‐316679’), Czech Ministries of Education and Health (Grant/Award Number: ‘NT13237‐ 4/2012, PRVOUK‐P26/LF1/4, RVO‐VFN64165/2012’).

## Conflict of Interest

None declared.
